# Displacement and hybridization reactions in aptamer-functionalized hydrogels for biomimetic protein release and signal transduction†
†Electronic supplementary information (ESI) available: Experimental methods and additional characterization figures. See DOI: 10.1039/c7sc03023a



**DOI:** 10.1039/c7sc03023a

**Published:** 2017-09-21

**Authors:** Jinping Lai, Shihui Li, Xuechen Shi, James Coyne, Nan Zhao, Fengping Dong, Yingwei Mao, Yong Wang

**Affiliations:** a Department of Biomedical Engineering , The Pennsylvania State University , University Park 16802 , USA . Email: yxw30@psu.edu; b Department of Biology , The Pennsylvania State University , University Park 16802 , USA

## Abstract

A variety of hydrogels have been synthesized for controlling the release of signaling molecules in applications such as drug delivery and regenerative medicine. However, it remains challenging to synthesize hydrogels with the ability to control the release of signaling molecules sequentially or periodically under physiological conditions as living cells do in response to the variation of metabolism. The purpose of this work was to study a novel biomimetic hydrogel system with the ability of recapitulating the procedure of cellular signal transduction and controlling the sequential release of signaling molecules under physiological conditions. In the presence of a small chemical, the signaling molecule is regulated to change from a DNA-bound state to a free state and the freed signaling molecule is able to regulate intracellular signal transduction and cell migration. Moreover, periodic exposure of the hydrogel system to the small chemical leads to sequential protein release. Since signaling molecules are important for every activity of the cell, this hydrogel system holds potential as a metabolism-responsive platform for controlled release of signaling molecules and cell regulation in various applications.

## Abstract 2

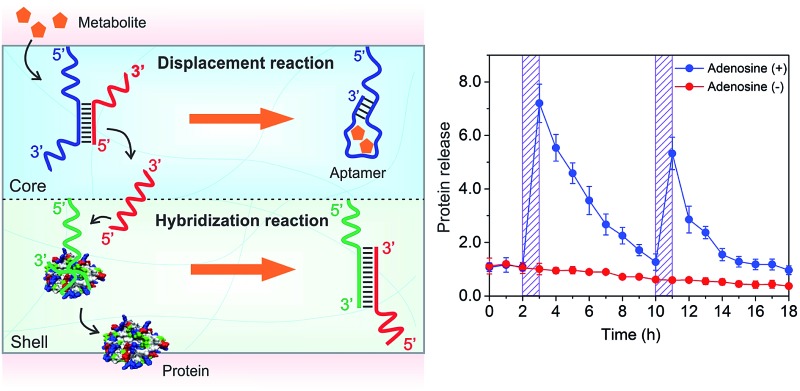
Combinatorial external and internal triggering events enable hydrogel to control protein release by mimicking signal transduction of the cell in response to metabolism.

## Introduction

Hydrogels have been widely studied for controlled release of various cargoes due to their biocompatibility and functional similarities to human tissues.^1^ For instance, the Tan group developed hydrogels that could release nanoparticles through fast gel–sol phase transition;^2^ the Willner and Liu groups developed hydrogels that were responsive to pH variation for cargo release;^3^ and our group recently synthesized a hydrogel that was responsive to exogenous oligonucleotides.^4^ However, currently available hydrogels release cargoes primarily based on the mechanisms of degradation, swelling, phase transition and/or exogenous triggering stimulation.^5^ While these mechanisms are promising for the development of hydrogels for numerous potential applications, it is challenging to apply them to achieve sequential or periodic release of signaling molecules in response to the variation of metabolism, which is greatly needed in biomedical applications. For instance, thyroid hormone and insulin needs to be delivered periodically for treatment of skeletal development or diabetes in response to the progress of tissue growth or the variation of glucose concentration;^6^ and vascular endothelial growth factor and platelet-derived growth factor BB (PDGF-BB) need to be delivered sequentially and periodically for treatment of cardiovascular ischemia.^7^ Thus, there is a great need to develop novel hydrogels that can mimick the releasing function of the cell.

Cells respond to and release signaling molecules during the variation of metabolism through a series of stepwise signal transduction. Moreover, the cells do not sacrifice their integrity during the procedure of signal transduction or significantly leak signaling molecules under a nontriggering condition. Thus, when needed, the release of signaling molecules from the cells can be repeated over multiple cycles. The ability to mimic this mechanism observed in living organisms would lead to broad applications such as drug delivery, regenerative medicine, and molecular biosensing. The purpose of this work was to explore a hydrogel system that can recapitulate the procedure of cellular signal transduction to control the release of signaling molecules in response to a small chemical. In particular, we applied the principles of DNA–DNA and DNA–protein interactions to develop the biomimetic hydrogel for controlled protein release.

## Results and discussion

**Fig. 1:**
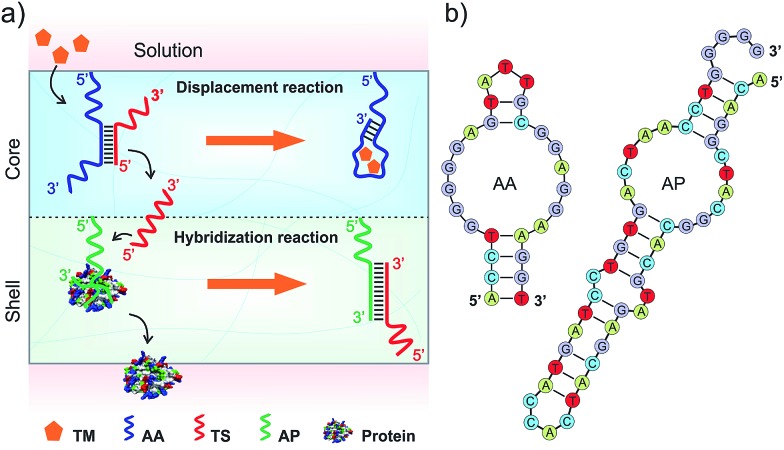
(a) Schematic illustration of regulating the DNA-bound and free states of protein *via* sequential DNA displacement and hybridization reactions. TM: triggering small molecule; AA: aptamer sequence binding to TM (*e.g.*, adenosine used herein as a model chemical); TS: triggering DNA sequence; AP: aptamer sequence binding to a target protein (*e.g.*, PDGF-BB). (b) Secondary structures of aptamers of adenosine (left) and PDGF-BB (right).

DNA strands form duplexes *via* Watson–Crick base-pairing hybridization reactions;^8^ moreover, the duplexes can undergo dissociation *via* strand-displacement (*i.e.*, exchange) reactions under physiological conditions.^9^ These hybridization and displacement reactions are fundamental molecular mechanisms observed in the biological systems and have been well studied from the view-points of thermodynamics and kinetics.^10^ The basic understandings of these reactions have allowed the use of DNAs as basic reactants to synthesize versatile materials such as molecular motors,^11^ DNA computers,^12^ catalysis machines^13^ and delivery systems.^14^ In addition to DNA–DNA interactions, basic studies have shown that DNA–protein interactions can be regulated by DNA. For instance, the states of transcription factors in binding to specific DNA sequences for transcription are regulated by nucleosomes in nuclei.^15^ Inspired by these biological mechanisms, we explored the hydrogel system to control the release of signaling molecules in response to a small chemical. The overall concept is shown in Fig. 1.

The hydrogel system was made of two compartments responsible for the displacement and hybridization reactions, respectively (Fig. 1a and Scheme S1, ESI†). The hydrogel was synthesized with polyethylene glycol (PEG) that has been demonstrated with good biocompatibility.^16^ The core compartment contained the AA–TS duplex; and the shell compartment contained the molecular complex made of AP and protein (Fig. 1a). These two compartments were both synthesized *via* free radical polymerization coupled with gas formation.^17^ During the polymerization, AA or AP was incorporated into each corresponding compartment. Adenosine and PDGF-BB were used herein as a model system to represent the small chemical (*i.e.*, TM) and the signaling molecule binding to AP, respectively. Their aptamers were previously selected and well studied.^18^

**Fig. 2:**
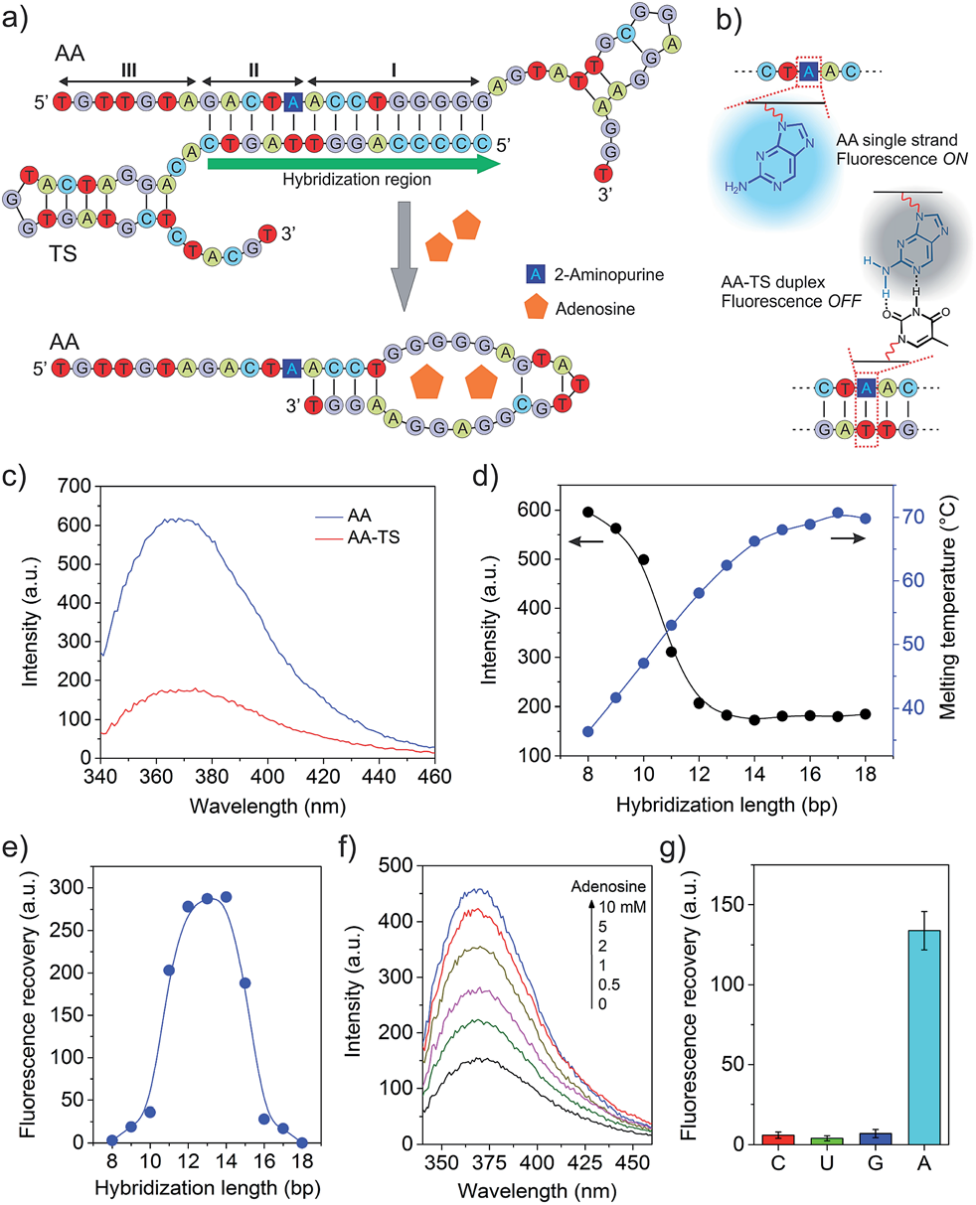
Examination of adenosine-triggered strand displacement. (a) Rational design of AA and TS sequences for the displacement reaction. The functional regions I and II in AA were designed to hybridize with the 5′-end of TS. The hybridization with 14 base pairs is shown here for clear legibility. (b) Structure of 2-aminopurine and schematic illustration of its fluorescence on/off status. (c) Fluorescence emission spectra of AA and AA–TS duplex in solution. Ex = 307 nm. (d) Effects of hybridization length on the fluorescence intensity and melting temperature of the AA–TS duplex (Em = 370 nm). (e) Relationship between fluorescence recovery and hybridization length of the AA–TS duplex in the presence of adenosine (5 mM). (f) Fluorescence emission spectra of the AA–TS solutions in the presence of adenosine. The number of base pairs was 14. (g) Fluorescence recovery of the AA–TS solution in the presence of nucleosides (1 mM). Error bars represent s. e. m (*n* = 3).

To develop the functional core compartment, it was important to ensure that AA and TS could form a stable duplex and meanwhile this duplex can be displaced in the presence of adenosine. A truncated adenosine aptamer was used to design AA with three functional regions (Fig. 2a). The first twenty-seven nucleotides at the 3′ end were the essential aptamer sequence for binding adenosine (region I); the following five nucleotides in the middle were complementary to one portion of TS (region II); and the seven nucleotides at the 5′ end were used to avoid potential steric hindrance of molecular interactions (region III). TS was designed to hybridize with one portion of AA and the entire sequence of AP (Fig. S1, ESI†).

We first evaluated the stability of the AA–TS duplex. AA was purposely modified with an internal 2-aminopurine since the fluorescence intensity of 2-aminopurine in a base pair can be significantly quenched in comparison to an unpaired form (Fig. 2b).^19^ By measuring the variation of fluorescence intensity, we were able to determine whether AA was in the form of single strand or helical duplex. As shown in Fig. 2c, the profile of free AA exhibits a strong fluorescence emission at 370 nm whereas that of the hybridized AA–TS duplex displays a significant decrease in the emission. A further study showed that the fluorescence intensity of the AA–TS duplex decreased with the increase of the number of base pairs (Fig. S1 and S2†). In particular, the fluorescence intensity sharply decreased when the number of base pairs was increased to the range between 10 and 12 (Fig. 2d). The fluorescence intensity reached plateau when the number of base pairs was increased to the range between 13 and 15. After this range, more base pairs did not further decrease the fluorescence intensity. The change in fluorescence emission is consistent with the analysis of melting temperature (Fig. 2d). The melting temperature quickly increases when the number of base pairs is increased from 8 to 14. Beyond 14 base pairs, the increase of the melting temperature is gradually slowed down. These results suggest that 13 to 15 base pairs would be a threshold to ensure the formation of a stable AA–TS duplex.

After the examination of the stability, we investigated the capability of adenosine in displacing AA from TS. The concentration of adenosine was fixed at 5 mM. A parabolic relationship between the number of base pairs and the enhancement of fluorescence intensity was observed (Fig. 2e and S3†). The enhancement of fluorescence intensity was gradually increased in the range between 8 and 10 base pairs and then sharply increased from 10 to 12 base pairs. The enhancement reached the maximal level when the number of base pairs reached the range between 12 and 14 base pairs. After that, it sharply decreased. This observation is reasonable due to the following reasons. When the number of base pairs was between 8 and 10, the AA–TS duplex had a very low stability and most AA strands existed in a free state. The addition of adenosine would not further increase the amount of free AA strands dramatically. Beyond this range, the duplex exhibited a high stability (Fig. 2d). The enhancement of fluorescence intensity would increase with the increasing amount of free AA only when TS could be displaced from AA. Therefore, these results show that adenosine could trigger the displacement of TS from AA. With the further increase of the number of base pairs over 14, the AA–TS duplex became more and more stable. It prevented adenosine from binding AA to induce TS displacement. Together, the data of stability and displacement demonstrate that rationally designed AA and TS could form a stable duplex whereas the two strands of the duplex could be displaced by adenosine. The TS sequence that formed 14 base pairs with AA was used in the following studies unless otherwise stated.

We further studied the effect of the concentration of adenosine on TS displacement. Fig. 2f shows that the fluorescence intensity increased with the increasing concentration of adenosine. In particular, it increased quickly when the concentration of adenosine was varied from 0 to 2 mM (Fig. S4†). At 2 mM, the efficiency of recovery was 43%. The intensity did not significantly increase over 5 mM. We also applied cytidine, guanosine, and uridine to treat the solution of the AA–TS duplex. The result suggests that TS displacement is adenosine-specific (Fig. 2g).

**Fig. 3:**
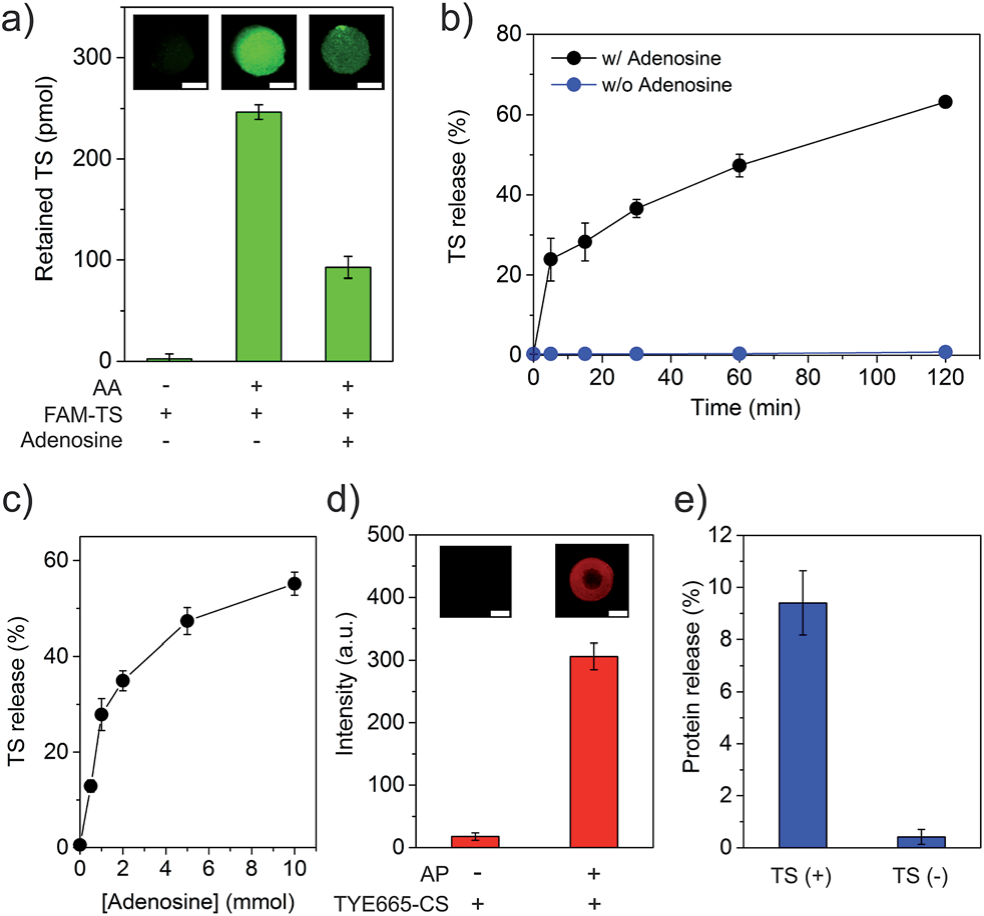
Evaluation of displacement and hybridization reactions in the hydrogels. (a) Fluorescence imaging of the core compartment to verify the displacement reaction. Scale bar: 2 mm (b) effect of displacement time on TS release in the presence of adenosine (5 mM). (c) Effect of adenosine concentration on displacement reaction. (d) Fluorescence imaging of the shell hydrogel stained with TYE665-CS. Scale bar: 5 mm. (e) Examination of TS-mediated protein release from the shell hydrogel. Error bars represent s. e. m (*n* = 3).

As demonstrating the displacement reaction in aqueous solutions, we further tested this reaction in the hydrogel. The AA-conjugated hydrogel was synthesized by copolymerization of Acrydite-AA with the monomers. After the treatment with FAM-labeled TS (FAM-TS), the AA-conjugated hydrogel displayed strong fluorescence whereas the native hydrogel exhibited very weak fluorescence (Fig. 3a). It demonstrated that AA and TS hybridized to form the duplex in the hydrogel. The treatment of the hydrogel with adenosine of 5 mM led to the significant decrease of TS retention (Fig. 3a). It demonstrated the ability of adenosine in displacing TS in the hydrogel environment. We also studied the time of displacement reaction on TS release. The results showed that the amount of TS displacement increased with time linearly (Fig. 3b). The percentage of displaced TS reached ∼47% and 63% in 60 and 120 min, respectively. We further studied the effect of the adenosine concentration on TS displacement. The reaction time for the adenosine-mediated displacement was fixed at 1 hour. In the absence of adenosine, less than 1% of TS was detected in the displacement solution (Fig. 3c). By contrast, the amount of TS displacement was ∼28% when 1 mM of adenosine was applied to treat the hydrogel. Moreover, the increase of the adenosine concentration led to the increase of TS displacement. The trend of the increase of TS displacement in the hydrogel *versus* the adenosine concentration was similar to that observed in the aqueous solution (Fig. 3c and S4†). The hydrogel was also treated with control nucleosides, and only 1.4%, 2.0%, and 1.4% of TS was released from the hydrogel that was treated with 1 mM of cytidine, guanosine and uridine, respectively (Fig. S5†). These results clearly demonstrate that the adenosine-mediated displacement reaction could occur in a hydrogel environment.

Following the characterization of TS displacement in the core hydrogel compartment, we studied the function of the shell compartment in protein sequestration and TS-mediated protein release. The AP sequence had two segments including the short aptamer truncated from its full-length parent sequence and a segment of AA (*i.e.*, ACCTGGGGG) at the 3′ end (Fig. S1b†). The reasons for copying this segment from AA and pasting it into AP are twofold. Firstly, it serves as an anchoring site of TS, resultantly enhancing the ability of TS in competing PDGF-BB for binding to AP and forming the TS–AP duplex. Secondly, with this molecular design, TS can be short. However, it is important to ensure that adding such a segment to a protein-bound AP does not sacrifice the binding affinity of the final sequence of AP. Thus, we evaluated AP incorporation and AP-mediated PDGF-BB sequestration. The incorporation of AP into the shell compartment was confirmed by the fluorescence staining of hydrogel with TYE665-CS that was complementary to AP (Fig. 3d). The results of PDGF-BB treatment showed that the incorporation of AP into the hydrogel led to 96% sequestration of PDGF-BB whereas the native shell (*i.e.*, without AP) could only sequester ∼1% (Fig. S6†). This remarkable difference showed that the AP-functionalized shell exhibited a strong ability of PDGF-BB sequestration. We further evaluated whether TS could induce the release of the sequestered PDGF-BB, by incubating the PDGF-BB-loaded shell in the TS solution for 1 hour. The result showed that the TS treatment led to 9.4% of PDGF-BB release, which was one order of magnitude higher than that without TS treatment (0.42%) (Fig. 3e). These data demonstrate that the rationally designed TS sequence had the ability of binding to AP *via* hybridization reaction after the formation of the AP–protein complex in the shell compartment.

**Fig. 4:**
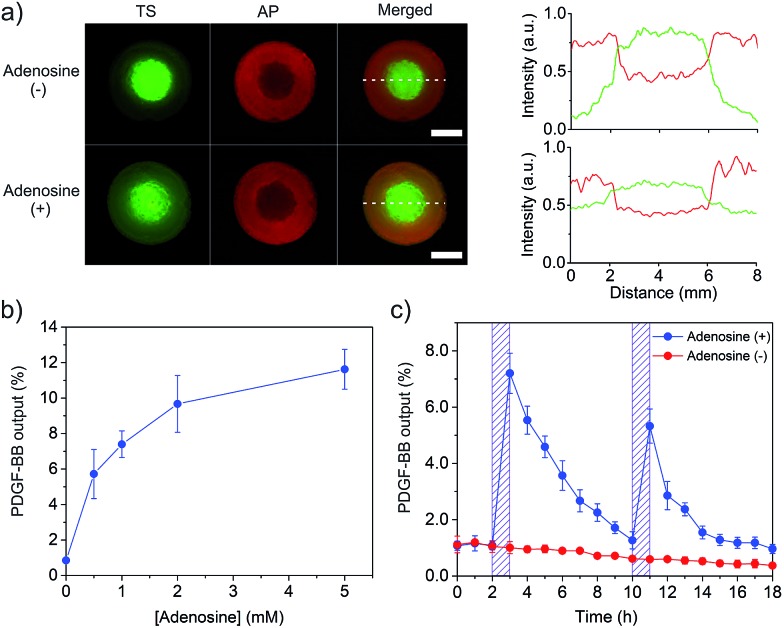
Displacement and hybridization reactions for PDGF-BB output from the hydrogel. (a) Fluorescence imaging of the core–shell hydrogel with (+)/without (–) adenosine treatment. The core was stained with FAM-TS (green, Ex/Em = 488 nm/540 nm) and the shell was stained with TYE665-CS (red, Ex/Em = 590 nm/650 nm). Fluorescence profiles along dotted lines drew in the merged images were also shown for clear comparison. Scale bar is 2 mm. (b) Effect of the concentration of adenosine on PDGF-BB output. (c) Hourly PDGF-BB output from the hydrogels treated with (+) or without (–) adenosine. In the (+) group, the hydrogel was treated with 1 mM of adenosine at two time points. Each time, the treatment last for 1 h.

After the demonstration of the displacement and hybridization reactions individually in the core and shell compartments, we assembled the two compartments to prepare the core–shell hydrogel (Scheme S1†). Its porous structure is shown in Fig. S7.† As it is important for TS to be captured by AP after TS displacement, we treated this hydrogel system with adenosine and observed TS distribution in the core and shell compartments under the fluorescence microscope. Strong green fluorescence signal of FAM-TS was observed in both core and shell compartments (Fig. 4a). By contrast, without the adenosine treatment, the green fluorescence signal mainly came from the core compartment. The semi-quantitative comparison of fluorescence intensities in different regions showed a significant decrease of green fluorescence intensity in the core compartment and a concomitant increase of fluorescence intensity in the shell compartment. Therefore, these results demonstrate that adenosine-displaced TS in the core compartment could be captured by AP in the shell compartment to form a TS–AP duplex *via* hybridization reaction.

With this information in hand, we further tested whether the combinatorial two reactions could lead to the conversion of a small chemical signal input (*i.e.*, adenosine) into a protein signal output (*i.e.*, PDGF-BB). The core–shell hydrogel was treated with a series of adenosine solutions. It showed that the PDGF-BB release increased with the concentration of adenosine (Fig. 4b). The release increased to 7.4 and 11.6% when the adenosine concentration was increased to 1 and 5 mM, respectively. The trend of PDGF-BB release (Fig. 4b) is consistent with that of TS release (Fig. 3c). Thus, these results demonstrated that the adenosine signal input could be converted into the PDGF-BB signal output *via* the displacement and hybridization reactions. It should be noted that the hydrogel system exhibited no significant change in the elastic modulus after protein release (Fig. S8†), which suggests that adenosine treatment did not change the bulk properties of the hydrogel system. We further studied the profile of the PDGF-BB release *versus* time. The hydrogel was exposed to adenosine at 2 and 10 h, respectively. The duration of each adenosine treatment last 1 h. The release media were collected hourly and replaced with a new release medium each time. As expected, the PDGF-BB was released periodically following the pattern of adenosine treatment (Fig. 4c). Notably, the release amount in the second cycle was lower than that in the first one. The hourly amounts of PDGF-BB release for the first and second triggering cycles were 7.2% and 5.3%, respectively. With more triggering cycles, the release amount was further decreased after each triggering event (Fig. S9†). This observation is reasonable because the total amount of PDGF-BB left in the hydrogel significantly decreased after each triggering cycle.

**Fig. 5:**
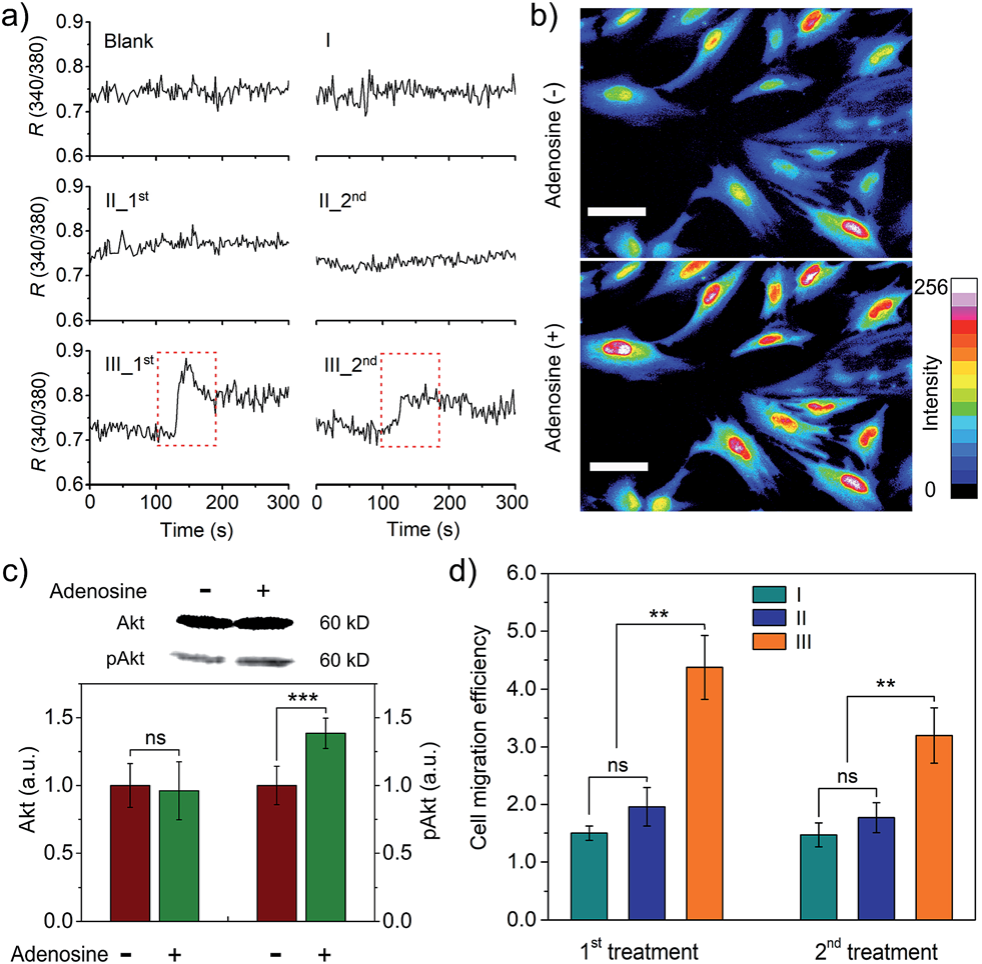
Signal output from the hydrogel for regulating intracellular signal transduction and cell migration. (a) Ratiometric fluorescence analysis of intracellular Ca^2+^ response using fura-2. Blank: without hydrogel or adenosine; Group I: hydrogel with adenosine (without PDGF-BB); Group II: PDGF-BB-loaded hydrogel without adenosine treatment; Group III: PDGF-BB-loaded hydrogel with adenosine treatment. 1^st^ and 2^nd^: SMCs were treated twice following the pattern of no or periodic PDGF-BB release. The time point of exposing the cells to the release medium was set at 0. (b) Fluorescence images of SMCs stained with Calcium Green™ before and after stimulation. The colors represent the intensity of fluorescence emission at 540 nm. Ex/Em = 488/540 nm. Scale bar: 50 μm. (c) PDGF-BB output for regulating pAkt expression in SMCs examined by western blot. Error bars represent s. e. m (*n* = 10), ***, *p* < 0.001. (d) PDGF-BB output for regulation of cell migration. See group information in (a). Error bars represent s. e. m (*n* = 3). ns = not significant; **, *p* < 0.01.

We further studied whether the output PDGF-BB can regulate signal transduction of smooth muscle cells (SMCs) at the levels of ions and proteins. Calcium ions serve as a second messenger in cells, playing important roles in cellular signaling.^20^ Thus, we first monitored intracellular calcium response to the extracellular PDGF-BB signal from the adenosine-triggered hydrogel using fura-2, a ratiometric fluorescent dye that binds to free intracellular calcium ions. Fig. 5a shows that SMCs in the control groups exhibited a negligible calcium response. By contrast, the cells in Group III (*i.e.*, adenosine-triggered PDGF-BB-loaded hydrogel) exhibited a strong calcium response. Moreover, the pattern of this calcium response (Fig. 5a) was consistent with the pattern of periodic PDGF-BB release (Fig. 4c). We further used Calcium Green™ to stain SMCs and captured cell images. In response to adenosine-mediated PDGF-BB output, a remarkable fluorescence enhancement was observed in the cells (Fig. 5b). It demonstrates the concentration of cytoplasmic calcium ions increased in response to the output PDGF-BB signal. In addition to the examination of signal transduction at the level of ions, we studied signal transduction at the level of proteins. P13K/Akt signaling pathway is important in regulating cell survival, proliferation, migration and differentiation.^21^ When this regulatory pathway is activated, Akt is phosphorylated and the concentration of phospho-Akt (pAkt) increases.^22^ Fig. 5c shows that the cellular level of pAkt increased owing to the phosphorylation of Akt in response to the PDGF-BB output from the adenosine-triggered hydrogel.

The outcome of regulating signal transduction pathways at the molecular levels will be eventually displayed by the cell behavior such as migration. PDGF-BB was a soluble signaling molecule that has been reported to regulate the migration of SMCs.^23^ As cell migration is essential for the development and maintenance of tissues and organs,^24^ a cell migration study was carried out to illustrate not only the outcome of signal transduction at the cell level but also the potential biomedical application of this hydrogel system. The results showed that adenosine-triggered PDGF-BB signal output from the hydrogel induced SMC migration, demonstrating that periodic exposure of the hydrogel to adenosine led to periodic PDGF-BB output for regulatory cell migration (Fig. 5d). To confirm the cell response resulted from the released PDGF-BB not adenosine, we also used free adenosine to treat the cells directly. The results showed that free adenosine with the concentration as used in our current experimental design did not induce the change of the cells as significant as the released PDGF-BB (Fig. S10† and 5d).

## Conclusions

In summary, we have demonstrated a biomimetic hydrogel system that has the ability of transforming a small chemical signal into a protein signal output *via* the sequential displacement and hybridization reactions under physiological conditions. The sequential out-in-out procedure of signal conversion does not need physical stimulations such as light and heat or the use of exogenous nucleic acids as molecular triggers, which makes the release control more effective and less complicated in response to metabolism. It is also important to note that while adenosine and its aptamer were used in this work, they can in principle be replaced with any metabolites (*e.g.*, glucose) and their corresponding aptamers. Similarly, PDGF-BB and its aptamer can be replaced with other signaling proteins (*e.g.*, insulin) and aptamers. Meanwhile, it is worthy of noting that the aptamer sequences after molecular design need to be carefully evaluated to ensure that they do not lose their ability of sequestering target proteins in the hydrogel. With rational design, this biomimetic hydrogel system would constitute a general platform of controlling the output of signaling proteins for versatile potential applications such as drug delivery, cell regulation, molecular sensing and regenerative medicine.

## Conflicts of interest

There are no conflicts to declare.
