# Supplementary material for: Displacement and hybridization reactions in aptamer-functionalized hydrogels for biomimetic protein release and signal transduction
Source: Chem Sci. 2017 Sep 21;8(11):7306–11. doi: 10.1039/c7sc03023a (PMC5672785; doi:10.1039/c7sc03023a)
Supplement: Supplementary file 1 [file SC-008-C7SC03023A-s001.pdf]

Electronic Supplementary Material (ESI) for Chemical Science.
This journal is © The Royal Society of Chemistry 2017

Electronic Supplementary Information

Displacement and Hybridization Reactions in Aptamer-functionalized Hydrogels for Biomimetic Protein Release and Signal Transduction

Jinping Lai,[a] Shihui Li,[a] Xuechen Shi,[a] James Coyne,[a] Nan Zhao,[a] Fengping Dong,[b] Yingwei Mao[b] and Yong Wang*[a]

[a] Department of Biomedical Engineering, The Pennsylvania State University, University Park 16802, United States.

[b] Department of Biology, The Pennsylvania State University, University Park 16802, United States.

1. Materials

All DNA oligonucleotides were purchased from Integrated DNA Technologies (Coralville, IA) and are listed in Table S1. Poly(ethylene glycol) diacrylate (PEG-DA, Mn = 700), Pluronic F-127, magnesium chloride, adenosine, uridine, guanosine, and cytidine were obtained from Sigma Aldrich (St. Louis, MO). Acetic acid, *N*, *N*, *N*', *N*'-tetramethylethane-1,2-diamine (TEMED), sodium bicarbonate, ammonium persulfate (APS), tween 20, phosphate-buffered saline (PBS), and sodium azide were purchased from Fisher Scientific (Pittsburgh, PA). Fluorescence calcium indicator Calcium Green™-1 AM and Fura-2 AM were purchased from Thermofisher Scientific. Platelet-derived growth factor BB (PDGF-BB) and its enzyme-linked immunosorbent assay (ELISA) kit were purchased from Peprotech (Rocky Hill, NJ). Bovine serum albumin (BSA) and 2, 20-azino-bis(3-ethylbenzothiazoline-6-sulphonic acid) (ABTS) were purchased from Invitrogen. All regents were used as received without any further purification. The PBS used in this project was supplemented with 5 mM magnesium chloride. The buffer used in this work was 140 mM PBS supplemented with 5 mM magnesium chloride and 0.09% sodium azide.

2. Methods

2.1 Analysis of DNA secondary structures

The secondary structures for DNA intermolecular hybridizations were predicted using NUPACK from website http://unafold.rna.albany.edu/?q=mfold/DNA-Folding-Form. The melting temperatures for DNA intermolecular hybridization were obtained using DNA Thermodynamics & Hybridization from IDT website http://biophysics.idtdna.com under the condition of 140 mM of sodium and 5 mM of magnesium.

2.2 Quantification of intermolecular DNA hybridization

The degree of intermolecular DNA hybridization was quantified *via* a 2-aminopurine-based fluorescence assay. The 2-aminopurine in a DNA sequence exhibits strong fluorescence when the sequence is under an un-hybridized state, but the fluorescence will be quenched when the sequence is under a hybridized state.[1] The 2-aminopurine modified AA (*AmPr*-AA) and various complementary sequences with different hybridization length (TS8-TS18, see Table S1) were dissolved in PBS with a concentration of 100 µM. All sequences were heated to 95 °C for 5 min and then gradually cooled to room temperature. To quantify the DNA hybridization, 10 µL *AmPr*-AA (100 µM) and 20 µL TS (100 µM) were transferred into a 96-well plate (Perkin Elmer, Waltham, MA) with 70 µL PBS. The mixture was incubated at room temperature for 30 min. Thereafter, the fluorescence emission spectrum of solutions was recorded by a Tecan M200 Pro Micro-plate reader (Tecan US Inc., San Jose, CA). The solution was excited at 307 nm and fluorescence emission was scanned from 340 nm to 480 nm. To quantify the DNA de-hybridization, adenosine, uridine,

guanosine, or cytidine was added to the *AmPr*-AA and TS hybridization solutions at room temperature with a concentration of 1 mM. After incubation for 1 hour, fluorescence emission of the solution was recorded using the same condition as hybridization. Fluorescence recovery efficiency was calculated with the following equation to describe the adenosine induced de-hybridization of AA-TS duplex:

$$Fluorescence\ recovery\ efficiency = \frac{Recovery}{Quenching} = \frac{F_{Ade} - F_{TS}}{F_{AA} - F_{TS}} \times 100\%,$$

where F_{AA} is the fluorescence emission of AA solution, F_{TS} is the fluorescence emission of AA-TS solution, and F_{Ade} is the fluorescence emission of AA-TS in the presence of adenosine.

2.3 Preparation of superporous hydrogels

Both core and shell hydrogel compartments were synthesized via free radical polymerization coupled with gas foaming reaction using a previously published method.[2] In brief, to prepare a cylindered core, 3.3 µL PEG700DA was mixed with 3.3 µL Pluronic F-127 solution (10% w/v) and 3.3 µL water with or without methacryl-modified DNA oligonucleotides (*Acryldite*-AA, 400 pmol). Next, 0.66 µL acetic acid, 0.44 µL TEMED and 3.3 µL APS (10 wt%) were added to the mixture. The final mixture (14.3 µL) was vortexed and quickly transferred to sodium bicarbonate in a cylindrical mold to initiate the formation of a superporous hydrogel. The procedure for preparing the shell was the same as that of the core with 7.6 µL PEG700DA, 7.6 µL Pluronic F-127 solution, 7.6 µL *Acryldite*-AP solution (60 pmol), 1.6 µL acetic acid, 1.1 µL TEMED, 7.6 µL APS, and a donut-like mold were used. After the synthesis, the hydrogels were thoroughly washed in deionized water for 24 h to remove any unreacted molecules.

2.4 Evaluation of AA conjugation in the core hydrogel compartment

The conjugation of AA in the core hydrogel compartment was verified by fluorescence staining and imaging. Hydrogel was stained with 100 pmol of *FAM*-TS in 100 µL PBS buffer at room temperature for 1 hour. After washed with fresh PBS, the hydrogel was imaged in a Maestro In Vivo Imaging System (Woburn, MA) using fluorescence mode (blue filter set: 445-480 nm bandpass filter for excitation; 515 nm longpass filter for emission).

2.5 Quantification of TS loading into and release from the core hydrogel compartment

FAM-TS was loaded into the core hydrogel compartment for the examination of adenosine-triggered TS release. Prior to TS loading, the hydrogel was dehydrated with tissue paper. Then, 80 µL of *FAM*-TS solution (400 pmol) was added to the dehydrated hydrogel that was incubated at room temperature for 10 h. Thereafter, the hydrogel was incubated in a 1 mL buffer for 1 h at 37 °C. The unbound *FAM*-TS in the supernatant was collected and quantified *via* fluorescence analysis. To detect the release of *FAM*-TS, the loaded core hydrogel compartment was incubated in 1 mL PBS at 37 °C. At predetermined time points, the

PBS solutions were collected and replaced with 1 ml of fresh PBS. To study the triggered-release of *FAM*-TS, adenosine, uridine, guanosine, or cytidine was added into the release buffer at predetermined time points. After release, the PBS solutions were collected and *FAM*-TS was quantified *via* fluorescence analysis.

2.6 Quantification of DNA concentrations

To quantify the *FAM*-TS in the release medium, the collected solutions were transferred into a 96-well plate. The fluorescence intensity was recorded by Tecan M200 Pro Micro-plate reader at 488 nm excitation and 540 nm emission. The concentrations of DNA solutions were determined with a standard curve.

2.7 Evaluation of AP conjugation in the shell hydrogel compartment

The conjugation of AP in the shell hydrogel compartment was also verified by fluorescence staining and imaging. Hydrogel was stained with 60 pmol of *TYE665*-CS in 100 µL PBS buffer at room temperature for 1 hour. After washed with fresh PBS, the hydrogel was imaged in a Maestro In Vivo Imaging System (Woburn, MA) using fluorescence mode (red filter: 580-610 nm bandpass filter for excitation, 640 nm longpass filter for emission).

2.8 Hybridization of TS to AP in shell hydrogel compartment

To study the hybridization of external TS with AP in the shell hydrogel compartment, 60 pmol *FAM*-TS in 1 mL PBS buffer was used to treat the shell hydrogel compartment. After incubation at 37 °C for a predetermined period of time, the PBS solution was collected and the unbound *FAM*-TS was quantified by fluorescence analysis to determine the amount of TS-AP hybridization in the shell hydrogel compartment. To study the adenosine-triggered release of TS from the core hydrogel compartment and its subsequent hybridization with AP in the shell hydrogel compartment, the core hydrogel was loaded with *FAM*-CS and the shell hydrogel was stained with 10 pmol *TYE665*-CS. The core and shell hydrogels were assembled and incubated in 1 ml PBS buffer at 37 °C for 1 hour. To trigger the *FAM*-TS release from the core, adenosine was added to the PBS buffer. The hybridization of *FAM*-TS released from the core with AP in the shell was monitored in the Maestro In Vivo Imaging System (Woburn, MA), and the hybridization amount was determined using the fluorescence intensity.

2.9 Characterization of PDGF-BB sequestration and release from the shell hydrogel

The shell hydrogel was gently dehydrated with tissue paper and then incubated with 200 µL solution of 100 ng PDGF-BB for 10 h. After the shell hydrogel was incubated with 1 mL of release medium (PBS supplemented with 5 mM magnesium chloride, 0.1% BSA, 0.05% Tween 20, and 0.09% sodium azide) at 37°C for 2 h, the release medium was collected to measure unbound PDGF-BB for calculation of PDGF-

BB sequestration. To examine the external TS-regulated PDGF-BB release, the PDGF-BB loaded shell hydrogel was incubated in 1 mL of release medium at 37 °C. At predetermined time points, the release media were collected and replaced with fresh release medium. TS was added to the medium at predetermined time points to trigger PDGF-BB release.

2.10 Preparation of the core-shell hydrogel system and examination of adenosine-regulated PDGF-BB output

TS-loaded core and PDGF-BB-loaded shell hydrogel compartments were assembled as illustrated in **Scheme S1** and incubated in 1 mL of release medium at 37°C. The whole release medium was collected and replaced with fresh release medium hourly. Adenosine was added into the release medium at predetermined time points. The adenosine treatment last for one hour each time. The time point for the first collection was set as time 0.

2.11 Quantification of PDGF-BB

PDGF-BB was quantified using a recombinant human PDGF-BB enzyme-linked immunosorbent assay (ELISA) (Peprotech, Rocky Hill, NJ). Prior to analysis, the collected samples were diluted with the diluent to ensure that the PDGF-BB concentrations fell within the detectable range. The absorbance of each sample was measured at 405 nm and subtracted at a reference wavelength (650 nm) using a Tecan M200 Pro Micro-plate reader (Tecan US Inc., San Jose, CA)

2.12 Measurement of the elastic modulus of the hydrogel system.

The elastic modulus of the hydrogel system before and after photoirradiation was measured using a universal testing machine (model 5966, Instron, Norwood, MA) at room temperature. The samples were tested under a crosshead speed of 1 mm/min. Compressive strain and stress were recorded and plotted as a stress-strain diagram.

2.13 Characterization of calcium response using fluorescence calcium indicators

To study the calcium response in smooth muscle cells (SMCs) under the stimulation with hydrogel, cells were first cultured in the low serum medium (*i.e.*, basic medium supplemented with 0.5% FBS) for 24 h. Thereafter, the cells were incubated with 10 µM Calcium Green^{TM}-1 AM in low serum medium with 0.1% Pluronic F-127 at 37 °C for 45 min, and at room temperature for 30 min. The cells were generally washed three times with low serum medium, and was placed in a fresh low serum medium supplied with 2 mM $CaCl_2$ for imaging. Fluorescence images of cell incubated with hydrogel before and after addition of adenosine were recorded on an Olympus IX3 inverted microscope using the FITC channel.

To study the calcium response in SMCs using Fura-2 calcium indicator, cells after starvation in low serum medium for 24 h was washed 3 times with Hank's Buffered Salt Solution (HBSS) with supplies (2 mM $CaCl_2$, 5.5 mM d-glucose, 4.2 mM $NaHCO_3$ and 0.1% BSA). Thereafter, cells were cultured with 20 μM Fura-2 AM in HBSS with supplies for 45 min at 37 °C, and for 30 min at room temperature. After washed with HBSS with supplies for 3 time, the cells were imaged under a Nikon eclipse TE2000 inverted microscope adapted with two UV light excitations at 340 nm and 380 nm.

2.14 Analysis of PI3k/Akt pathway regulation via Western blot assay

SMCs were seeded on 100 mm petri dish and were cultured with low serum medium for 24 h. Thereafter, cells were incubated with hydrogel with/without adenosine treatment for 3 h. Total protein of cells was extracted by a Mammalian Cell Lysis Kit (Sigma-Aldrich, US) based on the protocol provided by the manufacturer. The amount of total protein was determined by a BCA Protein Assay Kit (Thermo Fisher, US). 20 μg total protein was mixed with 2x Laemmli Loading Buffer (Sigma-Aldrich, US) and heated at 95 ºC for 5 min. Protein samples were subjected to 10% SDS-PAGE gel electrophoresis and transferred to a polyvinylidene difluoride membrane (PVDF, 0.2 μm, Bio-rad, US). The membrane was blocked with 3% BSA solution in TBST (0.1% Tween-20, 25 mM Tris, and 150 mM NaCl) and washed with TBST solution for 4 times. Then the membrane was incubated with anti-akt (Akt) primary antibody (1:500, Santa Cruz, US) at room temperature for 2 hours. After washing for 4 times, the membrane was incubated with HRP conjugated secondary antibody (1:1000, Santa Cruz, US) at room temperature for 1 hour followed by 4 times washing. Then membrane was soaked into Chemiluminescent Substrate (Thermo Scientific, US) for 5 min and imaged in a Biorad ChemiDoc XRS+ system. For phosphor-akt (pAkt) detection, membrane was treated with stripping buffer (Thermo Scientific, US), washed and blotted with anti-phospho-akt (pAkt) antibody using the same procedures to Akt.

2.15 Transwell cell migration assay

Prior to the cell migration experiment, smooth muscle cells (SMCs) were cultured in the low serum medium (*i.e.*, basic medium supplemented with 0.5% FBS) overnight. Similar to the PDGF-BB release study, the hydrogel was treated for 1 h with 20 μL 1 mM of adenosine solution at two different time points. After each adenosine treatment, the hydrogel was incubated with 750 μL low serum medium in a 24-well plate and the transwell insert with 2×10^4 SMCs was placed into the well. After 6 h of incubation, SMCs were stained with Calcein AM. The cells on the bottom side of the insert membrane were detected under a fluorescence microscope for cell counting. The wells containing only the low serum medium without hydrogel were used as control and the numbers of cells under migration in the hydrogel groups were normalized to that in this control group to calculate the cell migration efficiency. Statistical analysis was performed in Prisma 5.0 (GraphPad). One-way ANOVA followed by Bonferroni's Test was used to compare the cell number.

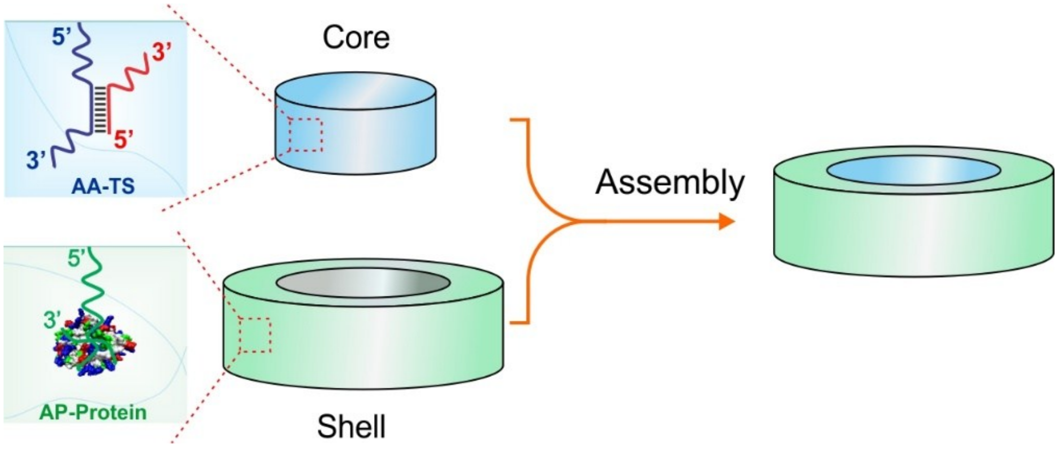

Scheme S1. Schematic illustration of the preparation of the hydrogel system.

Table S1. DNA oligonucleotides.

Name	Sequence
AmPr-AA	*5'*-TGTTGTAGACT/*i2AmPr*/ACCTGGGGGAGTATTGCGGAGGAAGGT-*3'*
Acrydite-AA	*5'-Acryd*/TGTTGTAGACTAACCTGGGGGAGTATTGCGGAGGAAGGT-*3'*
TS8	*5'*-GGTTAGTCACAGGATCATGGTGATGCTCTACGT-*3'*
TS9	*5'*-AGGTTAGTCACAGGATCATGGTGATGCTCTACGT-*3'*
TS10	*5'*-CAGGTTAGTCACAGGATCATGGTGATGCTCTACGT-*3'*
TS11	*5'*-CCAGGTTAGTCACAGGATCATGGTGATGCTCTACGT-*3'*
TS12	*5'*-CCCAGGTTAGTCACAGGATCATGGTGATGCTCTACGT-*3'*
TS13	*5'*-CCCCAGGTTAGTCACAGGATCATGGTGATGCTCTACGT-*3'*
TS14	*5'*-CCCCCAGGTTAGTCACAGGATCATGGTGATGCTCTACGT-*3'*
FAM-TS	*5'*-CCCCCAGGTTAGTCACAGGATCATGGTGATGCTCTACGT/*36-FAM*/-*3'*
TS15	*5'*-TCCCCCAGGTTAGTCACAGGATCATGGTGATGCTCTACGT-*3'*
TS16	*5'*-CTCCCCCAGGTTAGTCACAGGATCATGGTGATGCTCTACGT-*3'*
TS17	*5'*-ACTCCCCCAGGTTAGTCACAGGATCATGGTGATGCTCTACGT-*3'*
TS18	*5'*-TACTCCCCCAGGTTAGTCACAGGATCATGGTGATGCTCTACGT-*3'*
TYE665-CS	*5'-/TYE665/* CAGGATCATGGTGATGCTCTACGTGCCGTAGCCTGT-*3'*
Acrydite-AP	*5'-Acryd*/ACAGGCTACGGCACGTAGAGCATCACCATGATCCTGTGACTAACCTGGGGG-*3'*

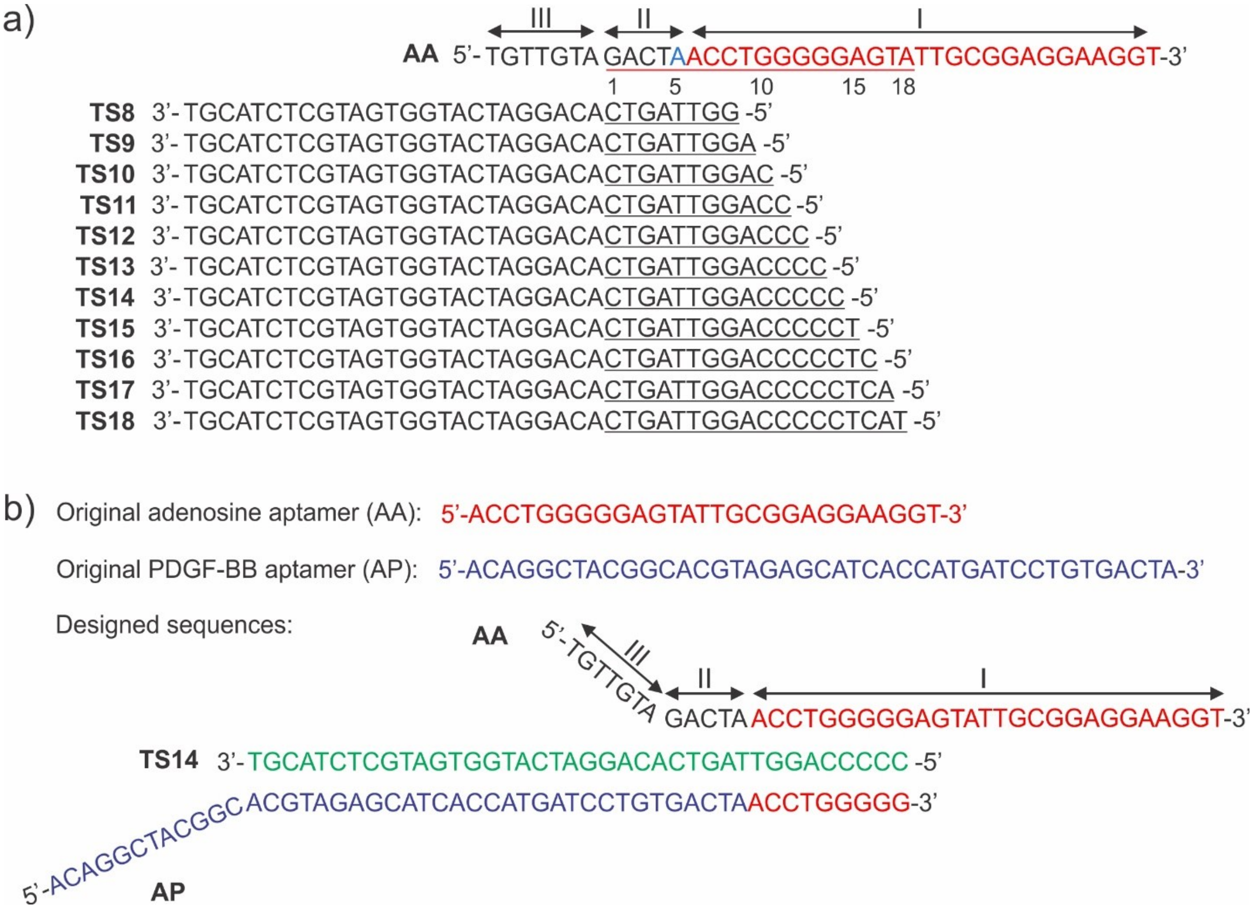

Fig. S1. (a) Illustration of the AA-TS hybridization with different length (underlined). Letter A (blue) indicates the 2'-aminopurine modification. Region I (red) shows the original sequence of the aptamer for adenosine. Region II and one part of region I were designed for AA-TS hybridization (underlined). Region III was a random sequence used as a spacer. (b) Sequences of originally truncated aptamers and the final sequences of AA, TS14 (typical example; most used in this work) and AP after molecular design. For clear legibility, the original adenosine aptamer is marked in red and the original PDGF-BB aptamer is marked in blue. The ACCTGGGG segment of AA is copied and pasted into AP for enhancing the TS-AP binding and making TS short as well.

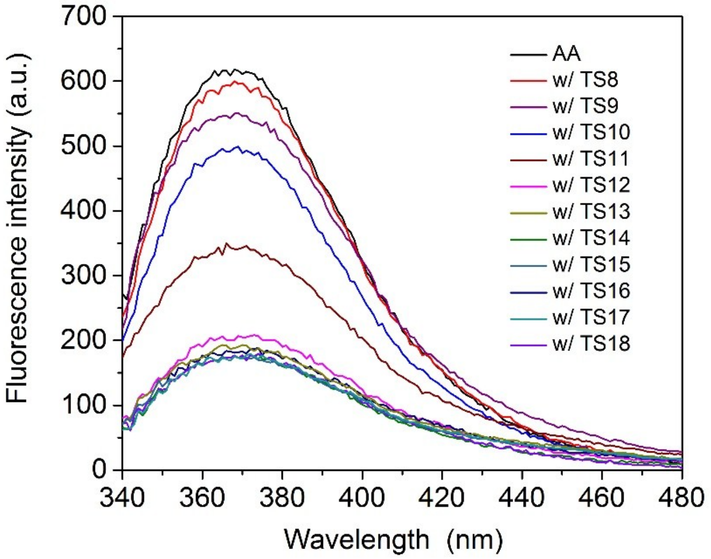

Fig. S2. Fluorescence emission spectrum of AA in the presence of TS of different length. Ex = 307 nm, [AA] = 10 μM, [TS] = 20 μM.

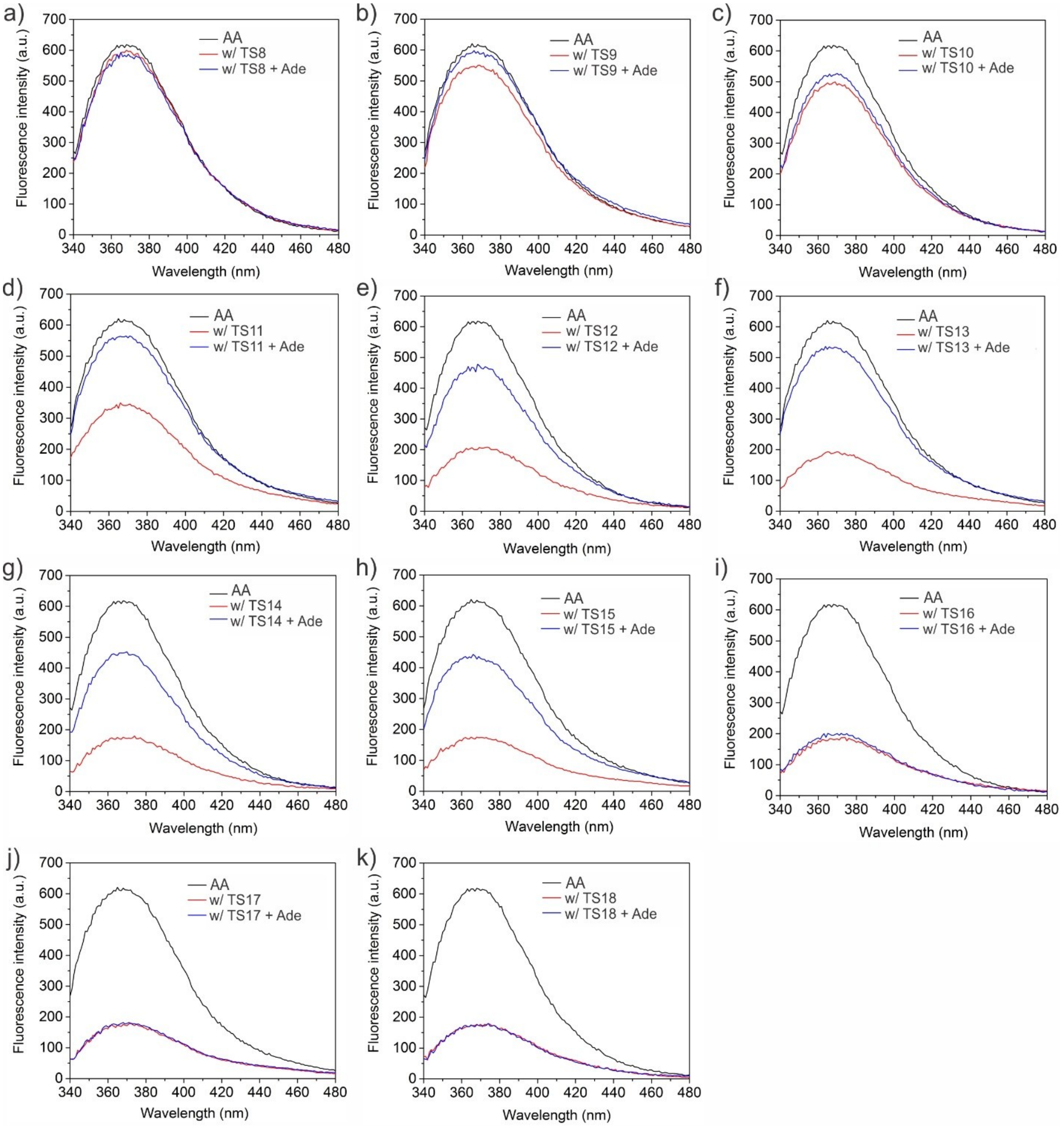

Fig. S3. Fluorescence emission spectrum of the AA-TS duplex with different hybridization length in the presence of adenosine. Ex = 307 nm, [AA] = 10 μM, [TSs] = 20 μM.

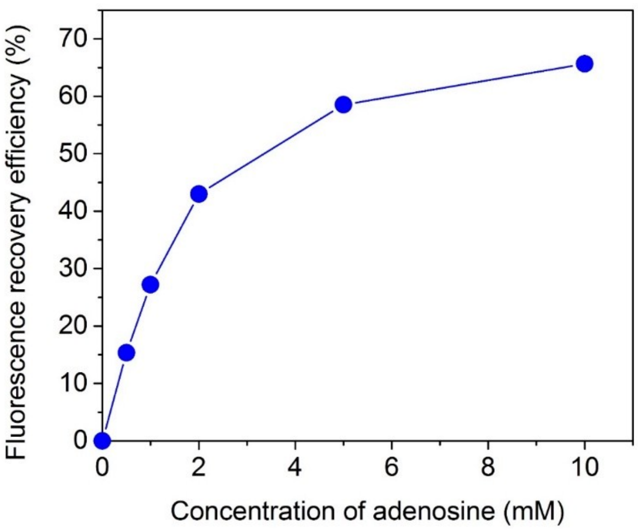

Fig. S4. Effect of adenosine concentration on the fluorescence recovery of the AA-TS duplex. [AA] = 10 μM, [TS14] = 20 μM, Ex = 307 nm, Em = 370 nm.

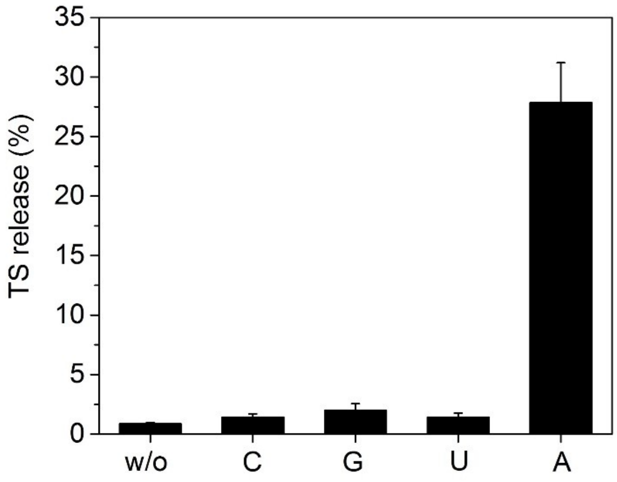

Fig. S5. Specificity of adenosine-induced TS release from the core hydrogel compartment. [C] = [G] = [U] = [A] = 1 mM.

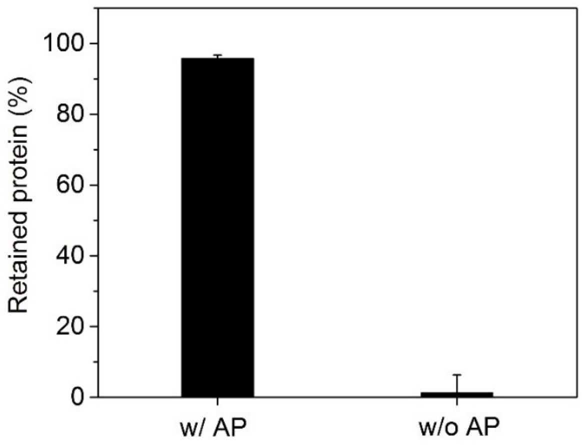

Fig. S6. Evaluation of PDGF-BB retention in the shell hydrogel compartment functionalized with/without AP.

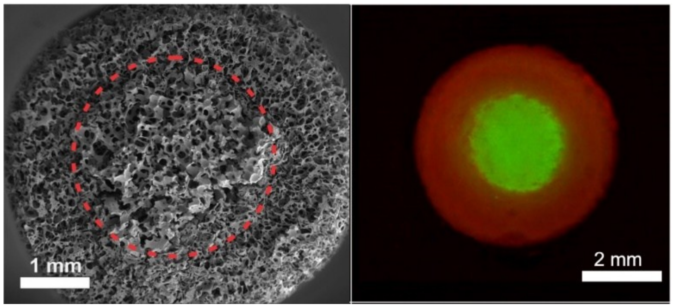

Fig. S7. SEM (left) and fluorescence (right) images of the core-shell hydrogel.

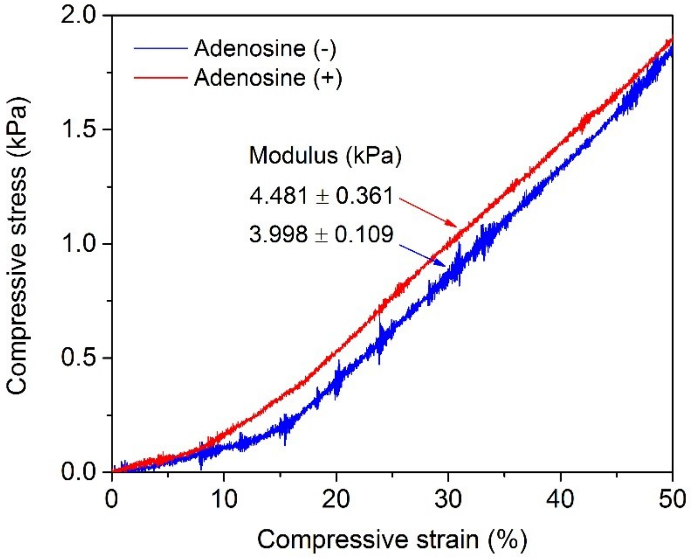

Fig. S8. Mechanical property of the hydrogel before and after adenosine incubation.

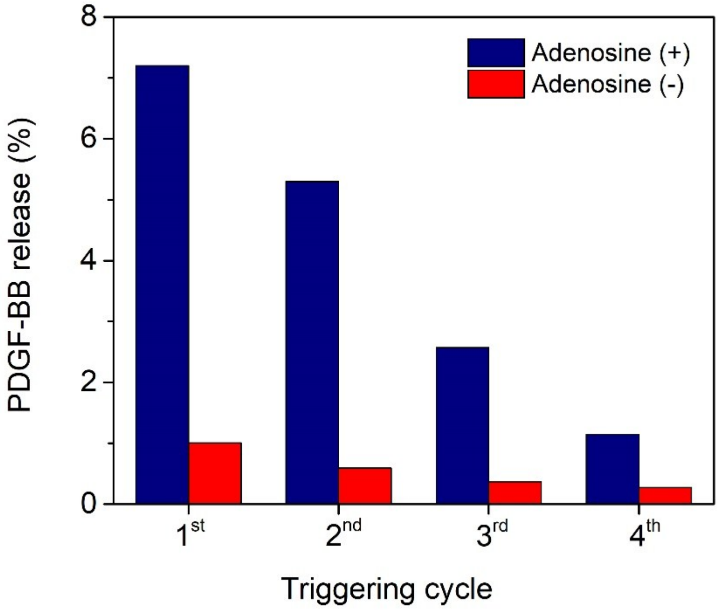

Fig. S9. PDGF-BB output from hydrogel after multiple cycles of triggering. [Adenosine] = 1 mM.

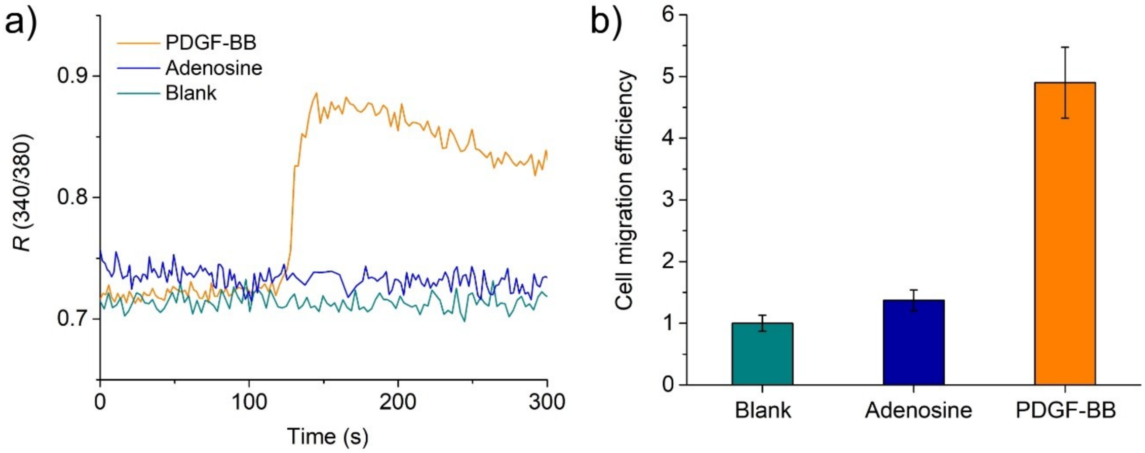

Fig. S10. Comparison of cell response to PDGF-BB and adenosine. (a) Ratiometric fluorescence analysis of intracellular Ca^{2+} response using Fura-2 in the presence of PDGF-BB (100 ng/mL) or adenosine (26.6 μM). (b) Cell migration. [PDGF-BB] = 10 ng/mL, [Adenosine] = 26.6 μM. Error bars represent s. e. m (n = 3).

References:

(1). E. L. Rachofsky, R. Osman and J. B. A. Ross, *Biochemistry*, **2001**, *40*, 946.

(2). R. A. Gemeinhart, H. Park and K. Park, *Polym. Adv. Technol.* **2000**, *11*, 617.
